# Challenges and opportunities with providing genetic testing and counseling for mucopolysaccharidosis type II in Kenya

**DOI:** 10.1186/s13023-025-03881-3

**Published:** 2025-11-04

**Authors:** Lucy N. Wainaina Mungai, Charles Njeru, Allan Njoroge, Michuki Maina, Syokau Ilovi, Ruth W. Nduati, Dalton Wamalwa, Beatrice Odongkara, Danny E. Miller

**Affiliations:** 1 Department of Paediatrics and Child Health, University of Nairobi, Nairobi, Kenya; 2 Department of Pathology, Aga Khan University Hospital, Nairobi, Kenya; 3 KEMRI Wellcome Trust Research Programme, Nairobi, Kenya; 4 Department of Internal Medicine, University of Nairobi, Nairobi, Kenya; 5 Department of Paediatrics and Child Health, Gulu University, Gulu, Uganda; 6 Department of Pediatrics, Division of Genetic Medicine, University of Washington and Seattle Children’s Hospital, Seattle, WA USA; 7 Department of Laboratory Medicine and Pathology, University of Washington, Seattle, WA USA; 8 Brotman Baty Institute for Precision Medicine, University of Washington, Seattle, WA USA

**Keywords:** Mucopolysaccharidosis type II (MPS II), Lysosomal storage disorder, Genetic counseling, Genetic testing, Kenya, Low- and middle-income countries (LMICs)

## Abstract

**Background:**

Limited or absent genetic counseling and testing resources in low- and medium-income countries lead to missed or late diagnoses for treatable metabolic conditions with irreversible complications. In some communities, misunderstanding about the etiology of a genetic condition may lead women whose children are affected to be viewed as a bad omen and become stigmatized or ostracized from their community. Mucopolysaccharidosis type II (MPS II), or Hunter syndrome, is a lysosomal storage disorder in which deficiency or inactivity of the enzyme iduronate-2-sulfatase leads to accumulation of glycosaminoglycans throughout the body. The diagnosis can be made through clinical assessment, enzyme activity analysis, or DNA sequencing. Treatment requires a multidisciplinary approach combining supportive care with disease-modifying therapies, including enzyme replacement therapy where available.

**Results:**

To understand the incidence and impact of MPS II in Kenya, we sought to provide counseling and genetic testing to individuals and families with suspected MPS II. After pretest counseling, we collected blood from 25 individuals to determine iduronate-2-sulfatase levels and sequence the *IDS* gene. We identified a pathogenic or likely pathogenic variant in 17 of 25 individuals and subsequently identified 18 female carriers in these families. We catalog the genotype of males with MPS II and correlate this with the phenotypic profile of these individuals, the female carrier rate, and mortality within the families.

**Conclusions:**

This study provides the first summary of genotype–phenotype correlations for MPS II in individuals from Kenya. These findings will allow the development of guidelines to identify individuals who may benefit from early evaluation, especially in those families where there is a risk of MPS II.

## Introduction

The worldwide burden of heritable genetic conditions is estimated to be about 5%, with about 90% of the 7.6 million newborns with congenital or inherited diseases living in low- and middle-income countries (LMICs) [1]. Genetic testing for these conditions is widely available in high-income countries and helps support disease diagnosis, inform treatment decisions, and provide accurate counseling about prognosis and recurrence risk [2]. Unfortunately, there is little to no access to genetic testing, genetic counseling, or a genetics provider to help patients or families navigate the psychological and medical impact of a genetic disease in many LMICs, such as Kenya [3], [1]. LMICs, as defined by the World Bank classification system, include nations with gross national income per capita between $1,086 and $13,205. Kenya, classified as a lower-middle-income country, faces significant disparities in healthcare infrastructure compared to high-income countries, with limited access to specialized genetic services and advanced diagnostic technologies.

Complicating factors to providing genetic testing and counseling services in countries such as Kenya are societal and cultural implications of sharing genetic information about heritable diseases with individuals or their families. For example, a study done to determine the community view of sharing a sickle cell disease diagnosis and trait information showed there was poor knowledge about the condition and denial among fathers who believed their wives were the cause of the condition [4]. These challenges extend to local communities, who may think of the mother of a sickle cell disease patient as “evil”, that her family had “evil spirits” that made her children sick, or that the condition was caused because of infidelity by the mother [4], [5]. These stigmata are complicated by cultural beliefs in which death may be viewed as a punishment, curse, or bewitchment, leading to mothers who have children that die of progressively debilitating conditions sometimes being viewed as the cause and then being sent away from their marital homes [6]. The lack of genetic testing, genetic counseling resources, and limited public awareness about genetics results in treatable metabolic conditions often being diagnosed late, after irreversible damage has occurred, with detailed information about disease etiology or recurrence risk not provided to families.

Mucopolysaccharidosis type II (MPS II), also known as Hunter syndrome, is an X-linked recessive disorder characterized by accumulation of glycosaminoglycans (GAGs), notably dermatan sulfate and heparan sulfate, in various tissues [7], [8]. Typically, these GAGs are physiologically degraded by the lysosomal enzyme iduronate-2-sulfatase (I2S), encoded by the *IDS* gene located at Xq28. Pathogenic variants in this gene lead to I2S deficiency with resulting GAG accumulation in tissues as well as increased urinary excretion. At the cellular level, GAGs accumulate in the lysosomes, eventually causing organomegaly [9]. Other findings in MPS II include coarse facial features, short stature, cardiac disease, respiratory disorders, and musculoskeletal abnormalities. MPS II has two main clinical forms: neuronopathic and non-neuronopathic. The neuronopathic form, which is more severe and affects about two-thirds of patients, is characterized by neurological involvement, primarily cognitive deterioration, and a more rapid progression of symptoms. Non-neuronopathic MPS II, on the other hand, involves minimal or no CNS involvement, with milder symptoms and a slower progression [10], [11], [12]. Individuals with MPS II will normally have a progressive debilitating course, although the rate of progression is also variable. Because MPS II is an X-linked condition, the phenotype is typically observed in 46,XY males who are hemizygous for the gene. However, there are reports of affected heterozygous females due to skewed X inactivation [13].

Diagnosis of MPS II is made by clinical assessment, analysis of enzyme activity, or gene sequencing. To identify carriers for MPS II, gene sequencing is needed. More than 600 *IDS* mutations linked to MPS II have been described [14]. The most common mutations are single nucleotide variants (missense or nonsense) (50%), small deletions (19%), and splice variants (9%)[15]. Establishing a genotype–phenotype correlation is challenging and not always consistent across different populations [16], [17], [18]. However, in general, frameshift, nonsense, splicing, and large structural variants (SVs; defined as insertions, deletions, or inversions larger than 50 bp) are typically associated with more severe disease, whereas missense variants are more often associated with more attenuated disease [19],[20]. Association between urinary GAGs and the degree of I2S deficiency and MPS II phenotype has also been described [16]. In the absence of treatment, individuals with attenuated disease may live into adulthood, while individuals with severe disease often die in the second decade of life [12].

Management of MPS II requires a comprehensive, multidisciplinary approach addressing the progressive, multisystem nature of the disease [21]. The foundation of care consists of supportive therapies including early surgical interventions for airway obstruction and joint contractures, aggressive treatment of respiratory infections, ensuring appropriate immunizations, educational support accommodations, and adaptive devices such as hearing aids for the nearly universal hearing loss [21]. Disease-modifying therapies, particularly recombinant human iduronate-2-sulfatase (I2S) enzyme replacement therapy, can provide additional benefits when accessible [21]. Given as a weekly intravenous infusion, I2S has demonstrated improvements in activity levels, quality of life, and reduction in organ damage, with increased survival compared to untreated patients [22]. However, I2S does not cross the blood–brain barrier and therefore does not address the behavioral or cognitive manifestations of the neuronopathic form [23, 24]. According to 2020 American College of Medical Genetics recommendations, all patients, including those with severe neuronopathic disease, may benefit from enzyme replacement therapy when available [25]. Data from randomized clinical trials demonstrated improvement in joint movement, quality of life, and reduced urinary GAG levels in patients receiving treatment compared to placebo [26]. These studies emphasized the importance of early diagnosis and timing of enzyme replacement therapy initiation. Effects of enzyme replacement therapy in adulthood did not show clear-cut benefits [27], [28] [29]. Other modes of treatment, such as substrate reduction therapy, which uses small molecule inhibitors to prevent GAG synthesis, have shown promising results and may be applicable to all MPS types [30], [31], [32]. Ongoing gene therapy studies in animal models have shown promise through reduced GAG levels in the brain and improved neurological outcomes [33]. Unfortunately, access to enzyme replacement therapy remains severely limited in LMICs, making supportive care the primary treatment modality for most patients in these settings. Some charitable programs do provide free enzyme replacement therapy for MPS II, Gaucher disease, and Fabry disease in select LMICs, emphasizing the importance of early diagnosis to maximize treatment opportunities.

In LMICs where facilities for genetic testing have not been established and there are no established newborn genetic screening programs, most individuals with treatable metabolic conditions like MPS II die before a clinical or precise molecular diagnosis is established. Studies on lysosomal storage diseases in these countries are either in the very early stages or are not being done. Recent work has highlighted the presence of the disease in certain communities in Kenya with shared ancestry [34], however the prevalence of the disease and carrier status in Kenya remains unknown because the lack of adequate screening and testing resources leads to underestimation of the true disease burden. Consequently, there is misdiagnosis of affected children and ongoing psychological distress in families who do not know the cause of this disease. This often leads to premature death of affected individuals and difficulty in providing accurate counseling to affected families [34].

Here, we aim to describe our experience with providing accurate genetic testing and counseling to individuals and their families affected by MPS II in Kenya through cases that provide a summary of the genotype–phenotype correlation of individuals with this condition. Our goal is to increase awareness of the benefits of early identification and counseling of individuals suspected to have MPS II or a family history of MPS II in Kenya. This will lead to an increase in the number of individuals referred for evaluation and care, and it reinforces the need for low-cost genetic testing in LMICs. The findings from this study will contribute to the development of guidelines to identify patients for early evaluation for MPS II and, eventually, for all treatable metabolic conditions in Kenya.

## Methods

### Study approval, recruitment, and counseling

Data for this series were collected in the endocrinology clinic at Kenyatta National Hospital (KNH), Kenya, which is the tertiary national referral and public hospital in the country. Ethical approval to perform the study and send the blood samples out of country for testing was obtained from the KNH/UoN research and ethics committee (PI40/02/2020).

Before blood samples were collected, mothers of individuals who had a phenotypic diagnosis of MPS II were given information about how an enzyme deficiency causes the condition. Parents and adult participants were asked to give written consent, and children 8–18 years of age gave assent. After confirmation of the diagnosis in male patients, the guardian of the patient was provided with an explanation of the diagnosis. Using diagrams, they were shown how MPS II is passed from the parent to the child. The study objectives were explained, and participants were counseled on the benefits of knowing the carrier state of female relatives. The parent of a child enrolled in this study underwent a brief interview to collect family history, draw a pedigree, and identify other contributing factors to the presentation. Participation was offered to siblings of affected males, and families were asked to bring other relatives who may have MPS II or be carriers of the condition. Relatives contacted through telephone came to the hospital to receive counseling about MPS II. After consent and pretest genetic counseling, participants were offered screening using a metabolic panel that included about 200 genes but were not offered exome or genome sequencing. All female adult participants recruited to the study consented to evaluation using the expanded panel of inherited metabolic disorders for their children.

For all individuals who consented to the study, a clinical examination to document the phenotype was performed. Extensive family history, especially on the maternal side, focusing on relatives who had similar phenotypic features or had died from a similar disease, was collected and pedigrees were drawn. Sixty individuals from 13 families were included in this series. Carrier screening was performed in seven families. Blood for molecular genetic testing for all 60 participants was collected on filter paper and shipped to a laboratory outside the country for testing.

### Molecular genetic testing and enzyme analysis

All molecular genetic testing and enzyme analyses were performed in a clinical laboratory (Centogene). Blood for molecular genetic testing for all 60 participants was collected on dried blood spot (DBS) cards using filter paper and shipped to the outside clinical laboratory. DBS collection was chosen as it is more feasible in resource-limited settings such as ours and does not require specialized storage or transport conditions, unlike whole blood collection which is typically only available in tertiary centers and requires rapid shipping. Panel-based sequencing (CentoMetabolic panel) was performed on an Illumina platform by performing a custom double stranded DNA capture approach to selectively enrich the coding regions, 10 bp of flanking intronic sequences, and known relevant variants beyond the coding regions for the 166 panel genes. Libraries were then generated with Illumina compatible adaptors and sequenced on an Illumina platform to obtain ≥ 20 × coverage depth for > 99.5% of the targeted bases. Variants were classified according to ACMG criteria [35]. Co-occurring pathogenic (P) or likely pathogenic mutations in other genes on the metabolic panel were documented. Testing of I2S enzyme levels was performed using fluorometric methodology (units, µmol/L/h). The assay’s limit of detection (LoD) was 0.8 µmol/L/h, and the reference was ≥ 5.6 µmol/L/h. The results of genetic and enzymatic analysis were accessed through an online portal provided by the laboratory testing company for provider access. Confidentiality was ensured during processing and handling of results. All participants underwent genetic counselling before the test results were given and further explanation done to clarify the genetic testing results. The male participants with pathogenic or likely pathogenic *IDS* gene variants were referred to the paediatric endocrinology clinic for follow up and possible inclusion in the enzyme replacement therapy charity program available in the hospital.

## Results

### Recruitment and counseling of families with MPS II

Study recruitment occurred through families of individuals with MPS II who were able to recognize features of this condition in children of other relatives or friends and through word of mouth with other healthcare professionals. Once identified, individuals and their families required substantial counseling regarding MPS II and the goals of our study prior to enrollment and sample collection. Challenges with counseling included discussing this condition with individuals and families who had lost multiple male relatives from what they initially considered a strange and unknown disease. In other cases, mothers who had been pushed out of their marriages because they were thought to be a bad omen were skeptical of the utility of genetic testing. For example, five of the 15 mothers of patients with MPS II were no longer married because of the associated male deaths in their family. For those mothers still in the same household, they reported that they were constantly reminded that they “had a problem”. Many of these mothers believed they had been “bewitched,” and that genetic testing or treatment could not change or explain that.

After discussion and counseling with a study team member, all participants that came to the hospital to discuss the study agreed to join the study. Recruitment of additional family members was sometimes limited by difficulty reaching female relatives living in rural areas to discuss participation in the study. After counseling, all 13 families referred for testing consented to having themselves and their sons join the study. An additional 35 female relatives from these 13 families agreed to be tested as well.

### Genetic testing results and clinical characteristics of individuals with MPS II

**Fig. 1:**
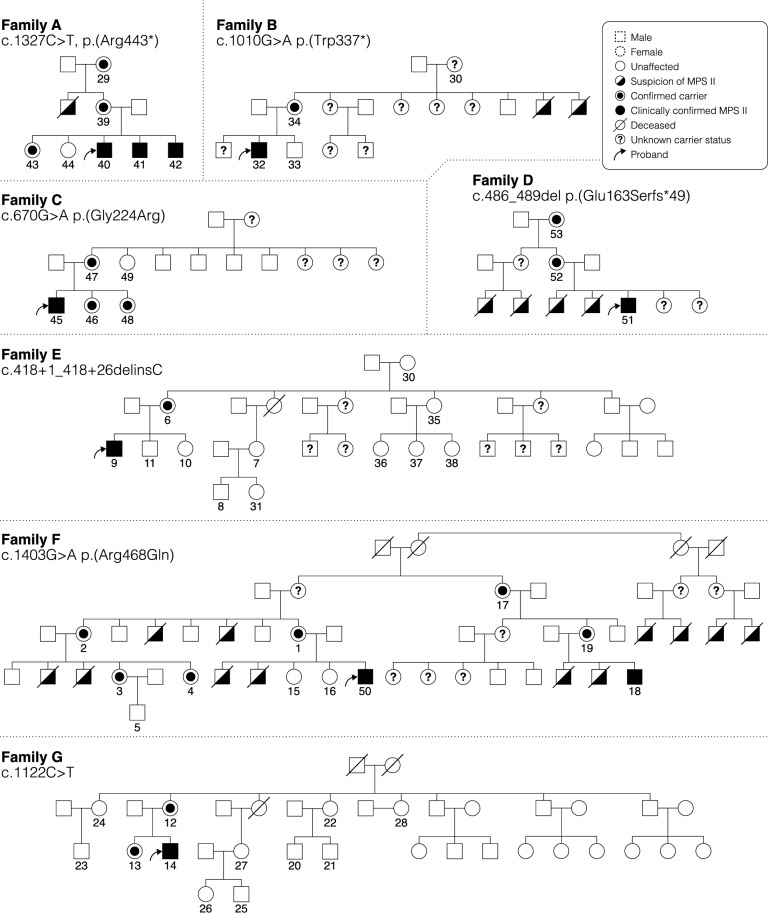
Pedigrees of select families with MPS II. Pedigrees illustrate the X-linked recessive inheritance characteristic of MPS II. Numbered individuals indicate those who participated in this study, and *IDS* variants identified in each family are noted.

Genetic testing was performed on all individuals with a clinical diagnosis of MPS II, first- and second-degree female relatives, and maternal male relatives under 10 years of age. In total, 60 individuals from 13 families were evaluated for *IDS* gene mutations, and pedigrees were obtained for 7 families (Fig. 1). Seventeen out of 25 male participants were confirmed to have pathogenic variants in IDS (Table 1) and 18 out of 35 female relatives were confirmed carriers of a pathogenic or likely pathogenic MPS II variant (Table 2). The eight male participants that did not have a pathogenic variant were all under 10 years of age and did not have features strongly suggesting MPS II. The average age of individuals confirmed to have MPS II by genetic testing was 8.8 years (range 9 months to 15 years). Among the molecularly confirmed cases, 7/17 were from three neighboring counties in Kenya that share linguistic and cultural practices. The others were distributed across five other counties. Out of the 17 confirmed MPS II cases, 12 had at least one additional family member evaluated for an *IDS* mutation after the diagnosis of the first male patient in the family (Table 2).

**Table 1:** Baseline clinical characteristics of 17 male individuals from 13 families with MPS II

	Attenuated MPS II (%)	Severe MPS II (%)
Total patients	6	11
Mean age in years at time of diagnosis	8.8	8.8
Variant type: deletion	1 (17)	6 (55)
Variant type: single nucleotide variant	5 (93)	5 (45)
*Characteristic*		
Attends school	6 (100)	0 (0)
Snoring	6 (100)	11 (100)
Dysostosis multiplex	6 (100)	11 (100)
Communicates Verbally	6 (100)	0 (0)
Macrocephaly	6 (100)	11 (100)
Female carrier in family	5 (39)	13 (72)
Other reported deaths from similar illnesses across the 13 families	4 (14)	25 (86)

**Table 2:** Correlation between phenotype and genotype of individuals with MPS II

Family	Confirmed cases of MPS II per family	Female carriers identified	Reported deceased male relatives	Variant identified by clinical testing	Variant classification*	Phenotype of proband
A	3 (siblings)	3 (grandmother, mother, sister)	1 (uncle)	c.1327C > T, p.Arg443*	P	Attenuated
B	1	1 (mother)	2 (uncles)	c.1010G > A, p.Trp337*	P	Severe
C	1	3 (mother, 2 sisters)	0	c.670G > A, p.Gly224Arg	P	Severe
D	1	2 (grandmother, mother)	4 (2 brothers, 2 nephews)	c.486_489del, p.Glu163Serfs*49	LP	Severe
E	1	1 (mother)	0	c.418 + 1_418 + 26delinsC	P	Severe
F	2 (cousins)	6 (mother, 2 aunts, 2 cousins, grandmother)	12 (2 brothers, 4 cousins, 2 uncles, 4 distant uncles)	c.1403G > A, p.Arg468Gln	P	Severe
G	1	2 (mother, sister)	0	c.1122C > T, p.Gly374 =	P	Attenuated
H	1	None tested	0	c.998C > T, p.Ser333Leu	LP	Severe
I	2 (cousins)	None tested	0	c.632del, p.Lys211Argfs*2	P	Severe
J	1	None tested	1 (brother)	c.253G > A, p.Ala85Thr	P	Attenuated
K	1	None tested	2 (uncles)	c.684del, p.His229Thrfs*51	P	Attenuated
L	1	None tested	0	c.418-2A > G	P	Severe
M	1	None tested	0	c.1010G > A, p.Trp337*	LP	Severe

^*^P: pathogenic; LP: likely pathogenic

Cases were classified as attenuated or severe MPS II based on clinical features and whether the individual was attending school and could communicate verbally. Clinical classification of MPS II severity based on cognitive function and school attendance has inherent limitations, particularly in LMIC settings where access to hearing support services, educational accommodations, and developmental assessments may be limited. Severe hearing impairment, as observed in several of our patients, can significantly impact educational performance and may lead to misclassification of cognitive abilities. Therefore, these phenotypic classifications should be interpreted with caution. Among 17 genetically confirmed MPS II patients, 65% (11/17) had a phenotype consistent with the severe form of the disease while 35% (6/17) had the attenuated form. Genetic testing identified 12 unique *IDS* variants in this cohort, meaning 12 different pathogenic variants were observed across the 17 confirmed cases. All variants identified have been previously reported in the literature. No de novo variants were identified in this cohort, indicating that all affected individuals inherited their pathogenic variants from carrier mothers, consistent with X-linked recessive inheritance. Single nucleotide variants (SNVs) resulting in either missense or nonsense mutations constituted 59%, while small deletions causing frameshift or splicing variants represented 24% (4/17) and 18% (3/17), respectively. Most attenuated cases (83%; 5/6) had a pathogenic SNV, whereas individuals with severe disease were more likely to have a deletion (55%; 6/11). Among the 17 cases of confirmed MPS II, three individuals were siblings (family A) and two pairs were cousins (families F and I). Two unrelated families (B and M) had the same c.1010G > A stop variant and both cases the probands had a more severe form of MPS II (Table 2).

Out of the 18 maternal female relatives who were found to be carriers for pathogenic or likely pathogenic MPS II variants, seven mothers, three sisters, three maternal grandmothers, three aunts and two cousins had the same genetic mutation as the proband in each family (Table 2). No de novo variants were identified in this cohort. From the family history, 29 maternal male relatives were identified who had died from a condition or phenotype similar to the proband from each of the 13 families. In three families (A, D and F), 17 male children with a clinical diagnosis of MPS II died from the disease (Fig. 1).

### Genotype/phenotype correlation of IDS variants

Out of the 13 families enrolled, 17 male participants had a pathogenic or likely pathogenic variant in *IDS*, and 18 female participants were confirmed carriers. All the confirmed cases were diagnosed late, with the youngest being 3 years of age and the oldest 17 years. There were 12 different genetic mutations identified across the 17 confirmed cases. All the mutations identified in this series have been previously reported.

Patients from families A, G, J and K presented with an attenuated form of MPS II. The six boys were mobile, had good language development, appropriate cognition for age, and all attended school. However, all were reported to snore at night, had short stature, and had partial to complete hearing loss. All had clawed fingers, thick skin, dysostosis multiplex, macrocephaly, coarse facial features, macroglossia, malocclusion in their teeth, hepatomegaly, and umbilical hernias. The oldest affected male from Family A had almost complete hearing loss, while another affected individual from Family A had good cognitive function and did well in school but relied on lip-reading to understand the teacher.

A c.1327C > T p.Arg443* mutation was identified in Family A, and biochemical testing confirmed low IDS function. This variant has frequently been reported as a common cause of MPS II, however, it is typically associated with a more severe form of the disorder, with onset of symptoms in early childhood, rapid progression of the disease, and a shortened lifespan. Zhang et al. investigated the molecular basis of MPS II in a Chinese population and reported that the p.Arg443* mutation comprised approximately 23% of all mutations identified and the clinical manifestation varied widely among affected individuals [36]. This is in contrast with our study, where the three siblings with this variant had an attenuated form of MPS II. The three affected individuals were in school and although the first two siblings had profound hearing loss, they were doing well in school. For example, one individual in this family (participant number 41) was reported to be the best in mathematics at his school. In Family A, an uncle died at 20 years of age with a reportedly similar phenotype.

In other cases, the severity of the disease was consistent with that observed in other studies. For example, affected individuals with the c.1010G > A (p.Trp337*) variant from families B and M had a severe form of MPS II, and in Family B, uncles who died in the first decade of life had features consistent with this condition (Table 2). That these individuals had severe disease is consistent with a study from the United Kingdom which found that the c.1010G > A (p.Trp337*) variant was one of the most common mutations observed in their cohort of patients, accounting for approximately 12% of all mutations, and that individuals with this variant tended to have the severe form of MPS II [7]. We also identified the c.670G > A (p.Gly224Arg) variant in Family C, where the proband in this family presented at 10 years of age with severe disease. While three female relatives were found to be carriers of the variant, there were no other reported deaths in the family with similar features as the proband. Whether there were other affected individuals is unclear as the culture of the community from where this family is from is one in which people were prohibited from talking about deceased family members. This variant has been associated with the severe form of MPS II in studies from both China [36] and Japan [37].

Overall, out of the 11 patients with severe MPS II, six were found to have pathogenic or likely pathogenic SNVs and five had an insertion or deletion variant (Table 2). A splice site variant found in Family D, c.418 + 1_418 + 26delinsC, affected the same intron–exon splice region (c.418 + 2 T > C) that has been previously described [38]. Finally, one variant identified in Family C, c.670G > A, p.Gly224Arg, has conflicting assertions of pathogenicity, with some asserting it to be likely pathogenic and others a variant of unknown significance [19], [39]. In our series, the proband exhibited severe disease and the mother and two sisters were confirmed to be carriers, thus this variant was annotated as likely pathogenic in our patient.

### Variants identified by expanded testing

Among the 17 molecularly confirmed MPS II cases, all were offered expanded genetic testing beyond the *IDS* gene. Families were counseled regarding this additional analysis, and all chose to proceed. Pathogenic or likely pathogenic variants in other genes were identified in 7/17 individuals (Table 3), with affected individuals carrying an average of two such variants (range: 1–8).

**Table 3:** Variants identified by expanded testing in individuals with MPS II

Family	Gene (OMIM #)	Variant identified	Zygosity	Phenotype
B, E, G, L	*G6PD* (305,900)	c.466A > G; p.(Asn156Asp)	Hemizygous	Anemia, congenital, nonspherocytic hemolytic, 1, G6PD deficient
D	*G6PD* (305,900)	c.466A > G; p.(Asn156Asp)	Hemizygous	Anemia, congenital, nonspherocytic hemolytic, 1, G6PD deficient
	*UGT1A1* (191,740)	c.1073del; p.(Asn358Thrfs*8)	Heterozygous	Crigler-Najjar syndrome types I and II; Hyperbilirubinemia, familial transient neonatal
	*ABCA1*	c.6083C > T p.(Ala2028Val)	Heterozygous	Tangier disease or familial HDL deficiency
	*AGL*	c.1885G > A p.(Glu629Lys)	Heterozygous	Glycogen storage disease type III
	*ATP7B*	c.1291 T > C p.(Cys431Arg)	Heterozygous	Wilson Disease
	*HGD*	c.919C > T p.(Arg307Cys)	Heterozygous	Alkaptonuria
	*PEX1* (602,136)	c.319G > A p.(Glu107Lys)	Heterozygous	Heimler syndrome 1; Peroxisome biogenesis disorder 1 A and 1B
	*PKLR* (609,712)	c.7 + 1G > T	Heterozygous	Anemia, congenital, nonspherocytic hemolytic, 2, pyruvate kinase deficient
F	*APOB* (107,730)	c.4210G > A p.(Val1404Met)	Heterozygous	Familial hypercholesterolemia; Hypobetalipoproteinemia
	*APOB* (107,730)	c.13654A > G p.(Met4552Val)	Heterozygous	Familial hypercholesterolemia; Hypobetalipoproteinemia
K	*BTD* (609,019)	c.1495C > T; p.(Pro499Ser)	Heterozygous	Biotinidase deficiency

Familial testing of the two *APOB* variants identified in Family F confirmed both variants were on the same haplotype

The most frequently detected variant on expanded testing was a hemizygous c.466A > G (p.Asn156Asp) change in the *G6PD* gene, located in the same cytoband (Xq28) as *IDS*. This variant appeared in 5 families (B, D, E, G, and L). While typically asymptomatic, *G6PD* variants can predispose individuals to hemolytic anemia under certain triggers, potentially complicating clinical management [40]. In Family D, additional variants were identified in *UGT1A1*, *BCA1*, *AGL*, *ATP7B*, *HGD*, *ITIH4*, *PEX1*, and *PKLR*, underscoring the potential complexity of overlapping metabolic and genetic conditions as well as counseling families about the results of expanded carrier testing in a population that is potentially not well represented in genetic databases.

In Family F, two missense variants in *APOB* (apolipoprotein B) were reported. *APOB* encodes a key structural component of low-density lipoproteins (LDL), which transport cholesterol throughout the body. Pathogenic variants in *APOB* can impair the normal clearance or metabolism of LDL particles, resulting in elevated cholesterol levels and hypercholesterolemia. Both variants are observed in population databases, but at low frequency. Familial testing confirmed that both variants were maternally inherited, and subsequent lipid testing confirmed that the proband in the family had elevated cholesterol, with reduced HDL and elevated LDL levels, putting him at increased risk of heart disease. While dietary counseling was provided as an immediate intervention, optimal management of familial hypercholesterolemia typically requires pharmacological therapy including statins, ezetimibe, and in severe cases, PCSK9 inhibitors or antisense oligonucleotides such as mipomersen. However, access to these advanced therapies remains limited in Kenya, highlighting the ethical considerations of expanded genetic testing in settings where identified conditions may not be readily treatable.

In Family K, a pathogenic variant (c.1495C > T; p.Pro499Ser) was detected in the biotinidase (*BTD*) gene, which is associated with autosomal recessive disease. While co-occurrence of MPS II and biotinidase deficiency may complicate phenotypic interpretation and highlights the importance of comprehensive genetic profiling, only a single pathogenic variant was identified in this gene. These findings demonstrate that expanded testing can reveal additional actionable genetic variants, providing an opportunity for broader clinical management and counseling.

## Discussion

This study represents the first and largest series to date in sub-Saharan Africa characterizing MPS II at the molecular level. Our findings confirm that the genetic and phenotypic spectrum observed in Kenyan patients largely aligns with reported variants and clinical presentations in other populations. Notably, both severe and attenuated forms of MPS II were identified, with severe disease often correlated with more disruptive (frameshift or nonsense) *IDS* variants. The presence of a similar range of genetic changes suggests that global molecular testing recommendations for MPS II may be applied to African populations. It should be noted that many commercial metabolic panels are primarily developed and validated using populations of European ancestry and may not capture the full spectrum of genetic variants present in African populations. This potential bias could lead to underestimation of disease prevalence or missed diagnoses in our study population. These panels may not include variants that are common or unique to African ancestry, potentially leading to false-negative results and underestimation of disease burden. The five families in our study carrying the *G6PD* variant c.466A > G (p.Asn156Asp) illustrate this point, as *G6PD* deficiency variants show significant population-specific distribution patterns. Future genetic testing initiatives in Kenya and other African countries would benefit from panels that include variants specifically identified in African populations or from more comprehensive sequencing approaches.

However, our work also highlights unique challenges in implementing genetic services in Kenya. The diagnosis of MPS II in Kenya is often delayed, with individuals typically identified only after families have experienced multiple male deaths. Cultural factors, such as the stigma attached to maternal inheritance and the notion that mothers are responsible for their children’s illness, can complicate the acceptance and understanding of genetic information. Mothers in our study frequently endured emotional trauma, including blame for the disease and, in some cases, estrangement from their marital homes. Although genetic testing was accessible through our study, a shortage of trained genetic counselors meant that the research team itself had to assume many of these responsibilities. This mirrors findings from other LMICs, where capacity building in genetic services often focuses on technical aspects over culturally appropriate counseling approaches [41]. Furthermore, beliefs in witchcraft or curses can undermine trust in genetic testing results, leading to doubt about diagnoses and the abandonment of treatment [1].

The absence of trained clinical geneticists or genetic counselors in Kenya further complicates communication of diagnoses and recurrence risks. In this study, we adapted available counseling techniques and involved local health professionals, but there is a pressing need for formal training programs in genetic counseling. Culturally sensitive, locally informed counseling frameworks would facilitate equitable genomic healthcare and support families during these emotionally challenging journeys. Furthermore, the identification of 18 female carriers in this study highlights the critical importance of reproductive counseling and the potential role of prenatal testing in family planning decisions. While prenatal diagnosis for MPS II is technically feasible through chorionic villus sampling or amniocentesis, implementation in Kenya faces multiple barriers including limited availability, high costs, lack of insurance coverage, and cultural considerations regarding pregnancy termination. In the Kenyan context, where pregnancy termination is legally restricted, prenatal testing would primarily serve to prepare families for the birth of an affected child and enable early intervention strategies. However, even this limited application could be valuable for families with known carrier status, allowing for immediate postnatal enzyme testing, early initiation of treatment, and comprehensive family planning counseling. Development of affordable, locally available prenatal testing services should be considered as part of a comprehensive genetic health strategy, even in settings where therapeutic options following positive results may be limited.

## Conclusions

Kenya is a highly diverse country, with individuals from over 40 ethnic backgrounds. This diversity makes it difficult to generalize the findings or recommend targeted variant testing for all suspected MPS II cases. Beyond MPS II, expanded genomic testing revealed additional variants in genes such as *G6PD* and *BTD*, some with potential clinical relevance. This underscores the value of broader genomic screening in resource-limited settings, where a single test can yield multiple actionable insights.

Ultimately, to improve outcomes for individuals with MPS II and other inherited conditions, there is a need for greater community education, early evaluation, and the establishment of newborn screening programs. We propose developing guidelines to help healthcare professionals recognize, counsel, and refer individuals with suspected genetic conditions. Initiating screening in families suspected of having inherited conditions and gradually expanding to a wider newborn screening program could be a practical approach. Integrating these efforts into the country’s Universal Health Coverage strategy would ensure more equitable access to genetic services.

## Data Availability

All data generated or analysed during this study are included in this published article.
